# Efficient Corona Suppression Coatings and Their Behavior in Corrosive and Icy Environments

**DOI:** 10.3390/ma18020254

**Published:** 2025-01-09

**Authors:** Kirill A. Emelyanenko, Maria A. Teplonogova, Alexandre M. Emelyanenko, Ludmila B. Boinovich

**Affiliations:** 1 A.N. Frumkin Institute of Physical Chemistry and Electrochemistry, Leninsky Prospect 31 bldg. 4, 119071 Moscow, Russia; ame@phyche.ac.ru (A.M.E.); boinovich@mail.ru (L.B.B.); 2 N.S. Kurnakov Institute of General and Inorganic Chemistry, Leninsky Prospect 31, 119071 Moscow, Russia; ma_teplonogova@igic.ras.ru

**Keywords:** coatings, superhydrophilicity, corona suppression, icephobicity, corrosion resistance

## Abstract

High-voltage transmission lines face significant challenges due to environmental exposure, including corona discharge, ice accretion, and corrosion, which impact their durability and operational efficiency. This study investigates the performance of hydrophilic and superhydrophilic organosilane coatings applied to high-voltage wires to address these issues. Using a combination of experimental setups simulating real-world conditions, we evaluated corona discharge losses, ice adhesion, and corrosion resistance on coated and uncoated wires. The results reveal that hydrophilic and superhydrophilic organosilane coatings offer a substantial reduction in corona discharge power losses, with a 25–60% decrease compared to bare wires. Additionally, the proposed hydrophilic coating exhibits ice adhesion characteristics similar to bare wires, in contrast to the higher ice adhesion observed for superhydrophilic samples. Corrosion tests further highlight the performance of the hydrophilic coating, which reduces corrosion currents by approximately threefold compared to bare wires, demonstrating enhanced protection and long-term stability. While superhydrophilic coatings offer some advantages in corona discharge reduction, their increased ice adhesion and higher corrosion rates limit their applicability. The hydrophilic organosilane coating thus emerges as the optimal tradeoff, balancing effective corona discharge mitigation, moderating ice adhesion, and enhancing corrosion resistance, making it a promising solution for improving the performance and longevity of high-voltage transmission lines.

## 1. Introduction

High-voltage transmission lines are the vital arteries of the modern power grid, carrying electricity over long distances, from generation facilities to urban centers and industrial hubs. However, their exposure to the open atmosphere presents several operational challenges, particularly concerning their durability and transmission efficiency. Among these challenges, corona discharge stands out as a significant issue due to its impact on energy consumption, safety, environmental health, and system performance [1,2,3,4].

The phenomenon of corona discharge can cause multiple operational problems. It manifests as a visible glow, and leads to the production of ozone, ultraviolet radiation, and the emission of audible noise, all of which pose health risks and contribute to environmental pollution. Furthermore, the energy dissipated in the corona discharge process translates directly into power losses in the order of W/m [5,6]. Considering the total length of high-voltage lines in the hundreds of thousands of kilometers, these losses present substantial economic burdens for utility providers and consumers [1]. In addition to these direct impacts, factors accompanying corona discharge aggravate issues related to corrosion [7,8,9,10]. Corrosion degradation not only shortens the lifespan of the transmission hardware, but also increases maintenance costs and the frequency of service interruptions.

Environmental factors intensify these challenges. Liquid and solid precipitations, like rain [11,12,13], high humidity [14], and frost and snow [15,16], which are common in many regions, increase the incidence and severity of corona discharges.

Multiple approaches have been proposed to mitigate power losses in high-voltage transmission, spanning various levels of the power infrastructure. These include the decentralization of power supplies and the use of smarter energy routing [17], as well as the deployment of underground or underwater cables [18], the application of insulating layers [19], modifications to the structural parameters of power lines [20], and active monitoring of overhead lines to lower voltage under adverse conditions. Although underground (and underwater) systems offer reduced environmental impact [18] and generally higher public acceptance [21], their high installation costs often make them economically unfeasible. Consequently, the total length of high-voltage lines constructed underground remains negligible compared with overhead lines, even in developed countries [22].

Adding insulation layers can help reduce certain transmission losses, but significantly increases conductor weight, and any defects in the insulating material may cause substantial corona discharge losses [19]. Hence, in practical applications, the most common strategy is to monitor real-time conditions on overhead lines to reduce voltage under the most severe weather or loading scenarios [23,24,25,26]. However, such monitoring-based approaches often limit throughput capacity exactly when energy demand is highest, which underscores the need for passive, cost-effective methods to curtail power losses in overhead transmission lines. As a result, there is continued interest in exploring economically viable technologies that can further reduce transmission losses while maintaining system reliability.

Corona discharge, that is, air ionization around the conductor, occurs when the electric field strength in the vicinity of the high voltage conductor is higher than air electrical breakdown limit. Under dry conditions, the coatings typically do not affect corona discharge significantly [5]. This is because, on the one hand, the electric field strength depends on conductor geometry, and, on the other hand, applied coatings are generally thin and do not change the geometry of the wire.

There are a lot of methods for characterizing anti-corona efficiency [27], which include the analysis of audio noise intensity [28,29], audio spectra [30,31], radio noise characteristics [32,33], corona discharge via UV photon counting [27] a UV-light camera [34], or measuring corona ignition voltage. However, the most direct method of assessment of the economic benefits of coating applications is based on measuring corona power losses [5,35,36,37]. Usually, these approaches are applied to two types of setups: cylindrical cages [33,38] and line/plane setup [5,36,39,40]. These setups allow creating around the conductor an electric field with the same strength as in power lines, to study corona phenomenon in conditions very close to real ones but on much smaller scale and in a more controllable environment.

The application of extreme wettability coatings, in particular superhydrophobic ones, with a contact angle above 150° and wetting hysteresis below 10°, and superhydrophilic coatings, for which materials include hydrophilic rough and/or porous materials on which aqueous media are completely spread or are soaked in, offers a promising solution to the issues faced by the power industry. At the same time, each of these coatings addresses the challenges associated with high-voltage transmission lines in unique ways [5]. So, for their effective long-term application in industrial conditions, a nuanced understanding of their functionalities under varying environmental conditions is essential. Not all coatings are universally applicable; the efficiency of a particular type of coating for long-term operation can vary dramatically depending on the climate, weather conditions, and specific operational demands of the transmission line [41].

Superhydrophobic coatings on wires, usually based on the application of both hierarchical surface morphology and low surface energy agents, such as fluorocarbons [5,42,43], oxysilanes [44] and hydrocarbons [10], are characterized by water-repellent properties, leading to droplets either bouncing or rolling off. This rapid removal of water decreases the period during which a droplet, being an element with high curvature, can induce corona discharge. As a result, while corona discharge may still occur during rain on superhydrophobic-coated wires, it tends to be short-ranged, significantly reducing the overall intensity of the discharge [5,42,43,44].

In contrast, superhydrophilic coatings, usually based on application of hierarchical roughness to intrinsically hydrophilic materials, like metallic oxide, without further chemical grafting [32,35,36,45], are characterized by rapid water impregnation inside the rough surface layer. Any excess water tends to accumulate as droplets hanging beneath the wire, which have a larger radius of curvature. These large and nearly flat droplets generate corona discharges at higher threshold voltages and produce less intense corona effects, although the discharge occurs over a longer period, due to the persistent presence of the droplets.

However, the applicability of different types of coatings is determined not by corona discharge performance alone, but also by their resistance to environmental degradation, long-term durability under operation, and proneness to ice accretion.

In colder climates, ice accretion on conductors is a major operational hazard [15]. Ice not only adds significant weight to the lines—risking physical damage from the increased load—but also serves as an initiation point for further corona discharges. Traditional methods of ice accretion mitigation, such as mechanical de-icing and heating [46,47,48] are energy-consuming and not always effective.

In our previous studies [5,40], we have demonstrated that both laser-processed superhydrophobic and superhydrophilic coatings can significantly reduce corona discharge. However, these coatings exhibit distinct limitations in long-term applications. Thus, superhydrophobic coatings slowly degrade under prolonged corona exposure, diminishing their effectiveness over time. Conversely, superhydrophilic coatings demonstrate improvement under conditions of exposure to both corona discharge and contact with water. However, the applicability of the superhydrophilic coatings studied earlier, in particular the laser-textured alumina surface without any grafted chemical layer, faces significant challenges. These include corrosion issues due to the lack of protective layers, high ice adhesion which can exacerbate ice accretion problems, and phase transitions in the aluminum wire’s surface layers during cyclic temperature variations in contact with water [49].

To overcome these limitations, this study investigates a new approach for imparting corona-protective properties by applying the hydrophilic and superhydrophilic organosilane-based coatings. This approach combines the beneficial properties of superhydrophilic surfaces in reducing corona discharge with the protective effects of organosilane layers against corrosion and ice accretion.

We evaluated these organosilane-based coatings under conditions that simulate real-world environmental exposure, focusing on their performance in corona discharge power loss reduction, ice adhesion strength, and corrosion resistance. By comparing them to both bare wires and previously studied coatings, we aimed to determine whether these new coatings offer a more balanced and durable solution. This study contributes to the field by addressing the specific limitations of earlier superhydrophilic coatings and introducing an alternative that could enhance the performance and longevity of high-voltage transmission lines.

## 2. Materials and Methods

### 2.1. Coating Preparation and Deposition

In this work, we examined the power losses due to corona discharge on 110 cm long samples of AC240/32 high-voltage wires that are commonly used in 220 kV power lines. The wires consist of a 32 mm [2] steel core surrounded by two layers of aluminum current carrier wires, totaling 26 wires and 240 mm [2] in cross-sectional area. The aluminum alloy 6101 of wires contains up to 0.7% Si, 0.7% Mg, 0.5% iron, 0.1% copper, and 0.2% other dopants, with the balance being aluminum. To remove preservative grease, all samples were cleaned first with water, then with ethanol. Three distinct sample types were investigated: bare (Bare), hydrophilic-coated (Phil), and superhydrophilic-coated (SPhil).

Bare samples were not subjected to any treatment after washing. Hydrophilic samples were subjected to dip coating in an organosilane solution, with the formation of a protective film after solvent evaporation. Superhydrophilic samples were first laser textured to create a hierarchical micro- and nano- morphology surface. Then, an organosilane coating was deposited onto the textured surface by the dip coating method. Laser treatment was performed using a nanosecond infrared laser (wavelength 1064 μm) equipped with a scanner head. This setup, in conjunction with a wire rotator, enabled the uniform treatment of the entire external surface of the wire. The treatment regime was based on a previously selected regime for flat alumina surfaces [50], which demonstrated enhanced mechanical strength. This improvement is attributed to laser-induced chemical modifications and the formation of a protective barrier layer of oxides and oxynitrides, which is characteristic of this treatment method.

The hydrophilic organosilane solution used for the preparation of Phil and SPhil coatings consisted of distilled water (300 wt. pt.), polyethylene glycol—400 (1800 wt. pt., Sigma Aldrich, St. Louis, MO, USA, base substance content of at least 91%), isopropyl alcohol (6000 wt. pt., Component Reagent, Moscow, Russia, base substance content 99.8%) and 3–aminopropyltriethoxysilane (600 wt. pt., “Silane”, Moscow, Russia, the content of the main substance 98.97%). All reagents were used as received, without additional purification. Deionized water, polyethylene glycol-400, and isopropyl alcohol were thoroughly mixed in the specified ratios. Subsequently, 3-aminopropyltriethoxysilane was added to the solution, which was stirred again. Samples were then immersed in the coating solution for a duration of 2 min, and were withdrawn at a constant meniscus withdrawal velocity of 20 mm/s. Finally, the coated samples were thermally cured at 120 °C for 60 min.

To summarize, Bare, Phil, and SPhil differ in surface morphology, which is smooth for Bare and Phil, and hierarchically rough for SPhil, and in the composition of the surface layer, thin alumina oxide film, organosilane film, and the thick textured alumina oxide layer covered by organosilane film for Bare, Phil, and SPhil, correspondingly (see Table S1).

### 2.2. Corona Discharge Losses Characterization

The prepared samples were evaluated using a high-voltage experimental setup at the Siberian Research Institute of Electric Power Industry [51]. The experiments were conducted using a wire-plane configuration, where the test samples were positioned horizontally, and suspended 112 cm above the measurement plane. The input voltage of *U*_test_ = 105 kV in the used setup caused an electric field strength around the wire, corresponding to that induced by the wire of industrial power lines, of 220 kV.

**Figure 1:**
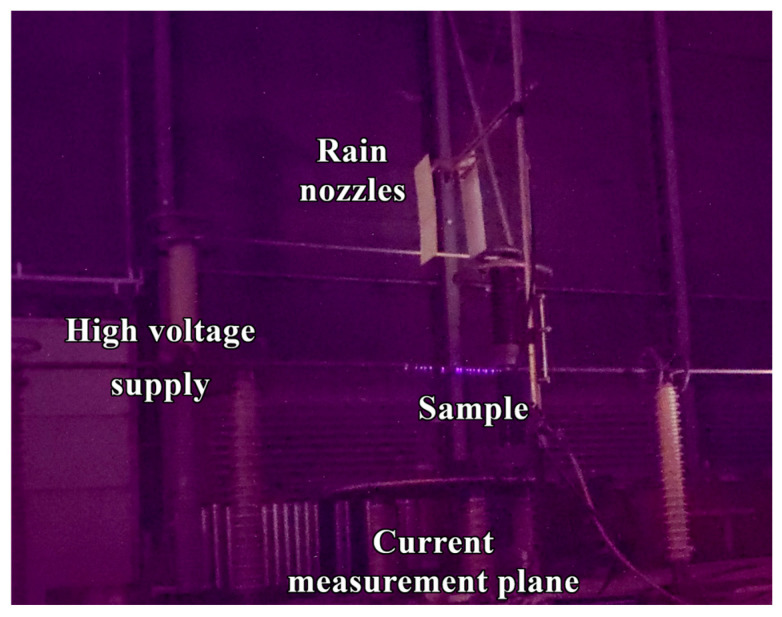
Photograph of the high-voltage corona discharge experimental setup. The experiment was conducted in near-complete darkness to enhance the effectiveness of electron-optical flaw detection. Consequently, the photograph was captured with a 30 s exposure, resulting in increased noise and reduced image quality, despite the use of a high-quality camera (Photographs under normal light conditions are given in Figure S1).

A photograph of the test equipment is given on Figure 1, whereas the electrical scheme and other details of the setup are discussed in detail elsewhere [5].

The samples were studied under both dry and simulated rainfall conditions. To create the rain conditions, a nozzle placed 150 cm above the wire was used to generate an even distribution of water droplets covering the entire test wire. The rainfall intensity was carefully controlled, ranging from 0.25 to 0.37 mm per minute on the wire surface, during each set of experiments.

The experiments were conducted by applying an alternating voltage with a frequency of 50 Hz to the wire samples. The voltage was measured using a standard capacitive high-voltage divider. Before conducting the rain tests, each sample was first evaluated under dry conditions to measure the relationship between corona loss power and the applied voltage. This measurement was performed over a voltage range from 0.8 × *U*_test_ to 1.3 × *U*_test_, with increments of 0.1 × *U*_test_.

Following this initial assessment, the corona power loss characteristics were measured under simulated rainfall. This was performed in cycles, where the first stage of each cycle involved measuring power losses across a voltage range from 0.5 × *U*_test_ to 1.1 × *U*_test_, also in steps of 0.1 × *U*_test_, lasting approximately 15 min. Immediately after this measurement stage, for each cycle the samples were subjected to a continuous application of *U*_test_ for 45 min (second stage). Once a two-stage cycle was completed, the next cycle commenced without delay. After three cycles, totaling 3 h of continuous corona discharge exposure, a final measurement of power losses across a voltage range from 0.5 × *U*_test_ to 1.1 × *U*_test_ was conducted.

To assess the longevity and self-healing processes of the coatings, as well as the effects of elevated voltage stress, the samples were left in an open atmosphere for 24 h after the first test session. Subsequently, an additional test under rain conditions was performed. This second test included two measurement stages within the same voltage range of 0.5 × *U*_test_ to 1.1 × *U*_test_, separated by 1 h exposure to 1.1 × *U*_test_ voltage.

The corona discharge phenomena were further investigated using an electron-optical flaw detector, which enabled the observation and differentiation of high-intensity discharges occurring at localized defects versus low-intensity discharges distributed uniformly across the wire surface. This allowed estimating the uniformity of corona inception centers along the wire, as well as determining the location of discharge centers (top, side, or bottom of the wire).

All measurements were conducted at a temperature of 21–24 °C, relative humidity of 53–65%, and 98,800–99,500 Pa atmospheric pressure.

### 2.3. Surface Morphology Characterization

Scanning electron microscopy (SEM) images were captured using a TESCAN AMBER GMH electron microscope (TESCAN, Brno, Czech Republic) at an accelerating voltage of 1 keV, with an Everhart–Thornley secondary electron detector. Energy-dispersive X-ray spectroscopy (EDS) was performed at 20 kV, using an Oxford Instruments Ultim MAX EDS detector (Abingdon, UK), calibrated with a cobalt standard. The EDS data were analyzed using AZtec 5.0 SP1 software.

The corrosion resistance of the wire with the developed coatings was analyzed based on the measurement of corrosion current. The corrosion current was calculated using the Tafel method, applied to potentiodynamic polarization curves. The measurements were performed in an 0.5 M NaCl aqueous solution using an electrochemical workstation, Elins P50× + FRA-24M (Elins, Moscow, Russia). The experiments were carried out in a three-electrode electrochemical cell (PAR K0235, Princeton, NJ, USA). In this setup, the working electrode was an aluminum sample, with or without a coating. A circular area of 1 cm^2^ was exposed to the electrolyte. A platinum mesh served as the counter electrode, and a reference electrode consisting of an Ag/AgCl electrode filled with saturated KCl solution was used. The sweep rate for monitoring potentiodynamic polarization curves was 1 mV/s.

### 2.4. Contact Angle Measurement

To characterize the surface wettability properties of the coatings used in this study, we measured the contact angles of deionized water on studied surfaces. Instead of direct measurements—which are inaccurate for hydrophilic and superhydrophilic coatings—we employed a captive bubble approach. In this approach, a 50 μL air bubble submerged in water touches the surface, with the surface positioned upside down. The contact angles of the bubbles were determined by capturing images of the bubble profile and applying Laplace fit optimization through digital processing. Correspondingly, the water contact angle was determined as 180° minus the bubble contact angle.

Contact angle measurements were conducted at 10 different surface locations for each sample. Each series of experiments was performed at least twice using different samples. The results presented in this manuscript represent averages of all performed measurements.

### 2.5. Ice Adhesion Measurements

Shear ice adhesion strength was measured using a centrifugal technique described in detail elsewhere [52]. The method utilizes a custom-made centrifugal device, where samples are mounted on a rotating disk within a climatic chamber to control the temperature and humidity conditions. Polypropylene bushings containing ice are attached to the sample surfaces, and the disk is spun at increasing speeds until the ice detaches. The centrifugal force at the moment of detachment is calculated based on the rotation speed and the mass of the ice, allowing for the determination of shear adhesion strength. This technique allows for the simultaneous testing of multiple samples, improving statistical reliability and accuracy in measuring adhesion strength by the direct comparison of different coating performances tested in parallel. The setup ensures precise control over the temperature, enabling the study of the impact of ice/solid contact aging on adhesion strength. Additionally, the use of stroboscopic illumination and synchronized video recording allows for accurate detection of the moment of ice detachment, ensuring precise calculations of the shear adhesion force.

## 3. Results and Discussion

As mentioned in numerous technical reports, under intense rain conditions, active corona discharge appears in wires below the nominal voltages, and rapidly grows upon voltage increase [43]. Under nominal voltage and at sea level, the bare wire for high voltage overhead conductors experiences corona discharge power losses of approximately a few W/m, which drastically increases with altitude [53]. That means substantial power and economical losses are on the line if no measures, like voltage decrease, are used. At the same time, wires with deposited hydrophilic and superhydrophilic coatings (Phil and SPhil) demonstrate corona discharge protection: both coatings demonstrate corona discharge losses 25% lower than that for of the bare sample. In particular, the data for Phil and SPhil coatings indicate the comparable corona discharge performances of these two types of coatings. Since all three samples, Bare, Phil, and SPhil, have contact angles below 90° (exact values are discussed below and presented in Table S1), the observed differences in corona discharge losses require more subtle analysis.

**Figure 2:**
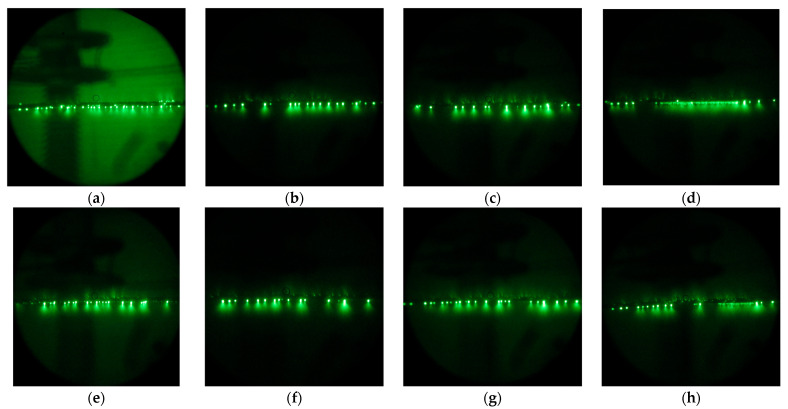
Electron-optical flaw detector images of Phil (**top row**) and SPhil (**bottom row**) wires in rain conditions under corona discharge at 1.1 × *U*_test_ upon increasing corona exposure time: just under the corona (**a**,**e**), after 3 h exposure to 1 × *U*_test_ (**b**,**f**), after 24 h resting period in dry conditions (**c**,**g**), after additional 1 h of 1.1 × *U*_test_ exposure in rain (**d**,**h**).

It is worth noting that, although the very clean aluminum surface demonstrates a contact angle of about 50 ± 3°, in practice, due to the high surface energy of aluminum, even brief atmospheric exposure leads to the adsorption of airborne contaminants, which increases the contact angle to nearly 90° or higher. At the same time, a hydrophilic organosilane coating demonstrates an initial contact angle of about 50.9 ± 0.8°, which tends to slightly decrease under contact with water due to the interaction of bound polyethylene glycol with water [54]. In turn, a superhydrophilic coating, due to its developed surface morphology, has an initial contact angle of 10.3 ± 0.5°, which preserves under continuous contact with water. Despite being hydrophilic in general, both organosilane coatings can be contaminated in an open atmosphere. That means that, during storage or exploitation in industrial conditions, due to the deposition of atmospheric dust or the adsorption of airborne organic contaminations, the surface of the coating may accumulate the droplet-pinning spots that impede free water flow on the surface over time. At the same time, when exposed to high concentrations of ozone and UV radiation characteristic of corona discharge, pinning spots formed by organic molecules are prone to burnout. The evolution of the density of these low wettability patches can be visualized by electron-optical flaw detector images showing the distribution of discharge streamers. Figure 2 shows streamers for both Phil and SPhil samples just exposed to corona (Figure 2a,e). These images indicate the presence of discharge centers on the top, side, and bottom parts of the wire. At the same time, upon prolonged exposure to corona, discharge centers migrate to the bottom part of the wire only (Figure 2b,d,f,h).

It appears that, rather than the average wettability of the hydrophilic surface, it is the density of pinning spots that plays a crucial role in influencing corona discharge intensity.

**Figure 3:**
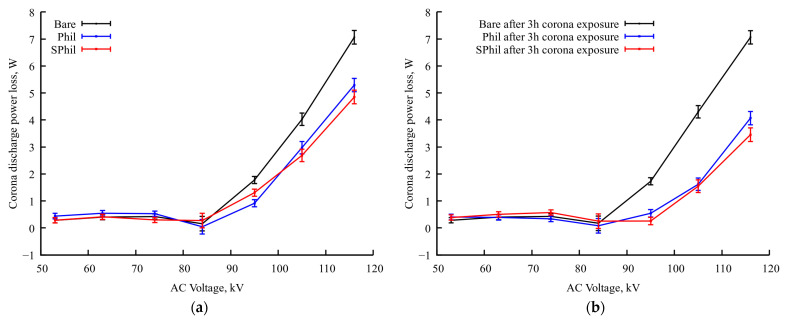
Dependence of corona discharge power losses on applied voltage in rain conditions (**a**) after test started (near zero total time of exposure to rain discharge conditions) and (**b**) after prolonged continuous exposure (3 h of exposure to rain discharge conditions).

Besides the redistribution of corona discharge centers, 3 h of continuous corona discharge exposure leads to a further substantial reduction in the corona current on Phil and SPhil samples (see Figure 3). The comparison of graphs in Figure 3a,b shows that the bare sample almost does not change its power losses characteristics, which corresponds to behavior described in the literature [5], and can be explained by the high wetting heterogeneity of a bare aluminum surface or, in other words, by a too high number of wetting defects. In contrast, corona discharge losses on Phil and SPhil coatings after 3 h of exposure to corona at a nominal voltage become ~2.5 times lower than that on a bare sample.

It is worth noting here that, the same way as with just under the exposure samples, after 3 h of exposition, Phil and SPhil coatings demonstrate corona discharge power losses very close to each other.

To check the proposed explanation of the role of airborne contamination, additional tests were conducted for both samples. The samples were subjected to a 24 h rest period in an open atmosphere, followed by 1 h exposure to rainy conditions with voltage elevated to 110% of nominal one. It should be noted that, as it follows from Figure 3, an increase in voltage from 100% to 110% leads to 2.5× corona intensity growth. Thus, an application of 1.1 × *U*_test_ voltage severely exacerbates the effects corresponding to corona exposure.

**Figure 4:**
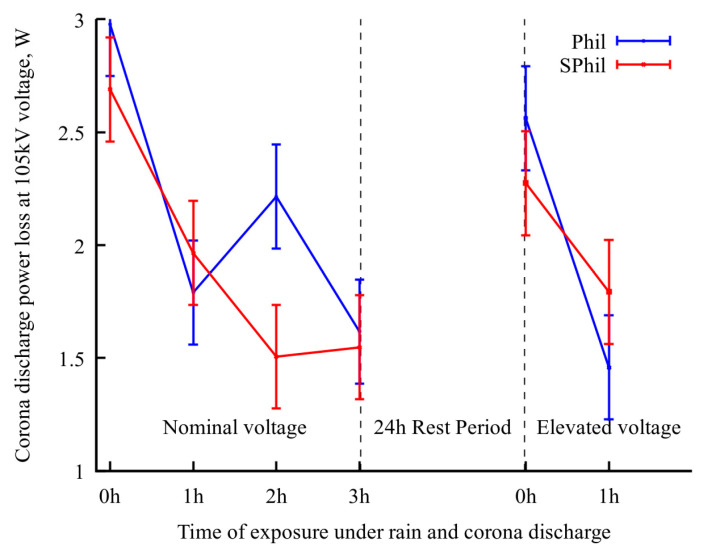
Evolution of power loss at nominal voltage upon exposure to different conditions. In the last period, the wire sample was exposed to 110% nominal voltage in between measurements.

As shown in Figure 4, a 24 h rest period leads to a rollback in corona suppression ability. Correspondingly, as can be seen from the electron-optical flaw detector images (Figure 2c,g), after a 24 h rest period, the discharge centers on the top and side parts of the wire were partially restored.

However, a subsequent 1 h of intense corona discharge again decreased the corona discharge current, and also decreased the density of the discharge centers on the top and side of wires. It is interesting to that the occurrence of continuous lines of discharges observed in Figure 2d,h, which resulted from the burnout of pinning spots on the bottom of the wire. This burnout allowed droplets to flow freely along the wire before detachment. As these droplets discharged during their movement, the flaw detector images captured over prolonged exposure times displayed this phenomenon as a continuous, semi-bright line.

**Figure 5:**
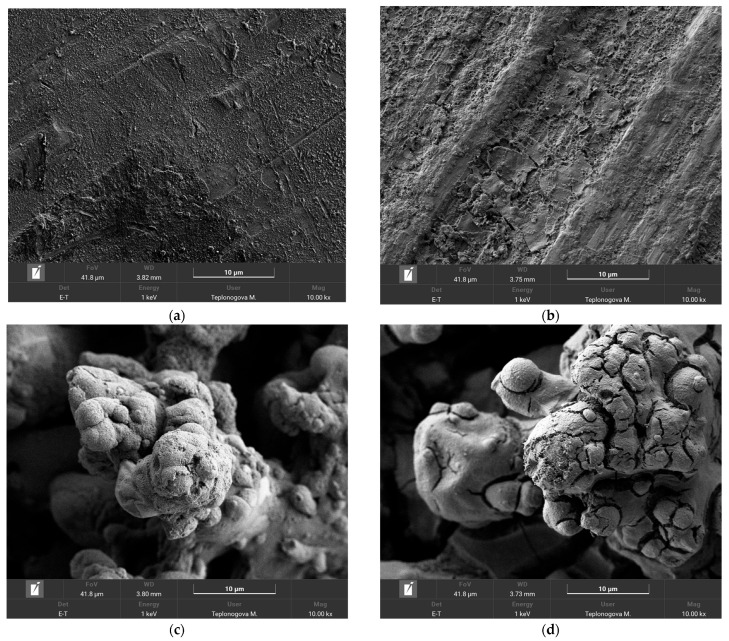
SEM images of Phil (**a**,**b**) and SPhil (**c**,**d**) coatings prior (**a**,**c**) and after corona discharge exposure (**b**,**d**).

To obtain more detailed information about the processes taking place on the wire surface during exposure to corona discharge, we have compared SEM images and the elemental composition of coatings before and after exposure to corona in rainy conditions. First, it is worth noting that the application of an organosilane coating does not change the morphology of the sample on microscale (Figure S2). As can be seen from the comparison in Figure 5a–d, the joint impact of running water and corona discharge causes the appearance of some peeling and cracks; however, this does not undermine the structural integrity of the coating. In addition, elemental maps of the surface after corona exposure, given in the Supporting Information, Figures S2 and S3, show the presence of the holeless layer of the organosilane coating on the surface, which allows for concluding coating preservation on top of both smooth and textured surfaces.

Joint analysis of the behavior of Phil and SPhil coatings under high voltage exposure in rain conditions, as well as SEM and EDX data (EDX element mapping and the composition table are given in the Supporting Information, Figures S3–S6), suggests the following mechanism of corona discharge suppression. The free flow of droplets on the surface allows water to form large hanging lenses beneath the wire, characterized by low curvature and, consequently, low corona discharge intensity. However, low-wettability patches that spontaneously develop on the wire surface, due to the adsorption of atmospheric contaminants, lead to the pinning of smaller droplets on the top and sides of the wire, resulting in increased corona power loss. Exposure of these patches to corona discharge causes them to burn out, subsequently restoring corona suppression capabilities. Notably, both adsorption and burnout are influenced more by surface energy and atmospheric contaminant composition than by coating wettability. Although the wettability of Phil and SPhil coatings differs significantly because of their different morphologies, their surface free energy remains the same, due to the presence of identical organosilane material in their surface layers. The latter finding was supported by EDX data (EDX element mapping is given in the Supporting Information, Figures S3–S6), which remained intact throughout the testing for both coatings. This shared characteristics account for the very similar evolution of corona power losses observed for both Phil and SPhil coatings.

The obtained results have not only shown a significant perspective on the application of hydrophilic organosilane coatings for combating the issue of power losses during transportation, but have also demonstrated that coatings which are simpler in fabrication, in particular the Phil coating, have comparable corona discharge suppression abilities. To be industrially viable, wires must also exhibit resistance to atmospheric icing and corrosion, as discussed in the Introduction.

To study the proneness of the proposed coatings to ice accretion, we have studied shear ice adhesion strength at *T* = −3 °C. The conditions with temperatures slightly less than 0 °C and high humidity are revealed to be the most dangerous during freezing rain, when the rapid icing of power lines takes place. Shear ice adhesion strength was evaluated by measuring the angular velocity at which ice detaches from flat, smooth, and textured samples mounted on a centrifuge disk. This setup allows for a statistically reliable comparative study of ice adhesion across various coatings, by enabling the simultaneous testing of up to 24 samples, aided by strobe lighting and video recordings. Adjusting the angular acceleration facilitates the investigation of different loading conditions, while varying the exposure time at a constant negative temperature enables the examination of ice relaxation effects. These features help to simulate real-life icing scenarios and assess the anti-icing performance of the coatings under diverse conditions. In this study, we analyzed the susceptibility of the coatings to icing by examining shear adhesion strength under three regimes: slow shear load increase, fast load increase, and prolonged equilibration at the target negative temperature, followed by a rapid load increase.

Data reported in Table 1 demonstrate that, for all testing regimes, Bare and Phil coatings have similar adhesion strength, which differs less than the statistical error. In turn, SPhil coatings demonstrate much higher adhesion to ice. It is worth noting that the detachment of ice from the SPhil coatings was accompanied by both adhesive and cohesive failure. It also worth noting that, unlike superhydrophobic coatings [52], no statistically significant decrease in adhesion during prolonged relaxation at negative temperatures is observed.

As discussed in the literature [55], the hydrophilicity of coatings has two opposing effects on ice adhesion strength. On one hand, the stronger binding of water molecules to the hydrophilic coatings by hydrogen bonding increases the adhesion. On the other hand, this hydrogen bonding promotes the formation of a quasi-liquid water layer (QLL) [55], which acts as a slippery layer, and thus aids in the detachment of ice. The significance of this latter effect is most pronounced when the thickness of the QLL exceeds the surface roughness.

For the Sphil coating, which features a highly developed surface morphology, the reduction in ice adhesion associated with the QLL is minimal. Instead, the enhanced affinity to water and the increased contact area due to surface roughness significantly elevate ice adhesion. Interestingly, the measured ice adhesion strengths of the Phil and Bare samples fall within the margins of measurement error, suggesting that the effects of the QLL and the increased binding of water molecules to the surface either offset each other or are negligible.

From the perspective of atmospheric icing resistance, the Phil coating exhibits greater attractiveness than the SPhil, as it does not enhance ice adhesion relative to the untreated sample.

Another important factor that determines the applicability of the coatings for power lines is corrosion, which may endanger both the structural properties and the conductivity of the wires. To assess this factor, the evolution of corrosion current upon immersion of coatings into the 0.5 M aqueous solution of NaCl was studied. In addition to Bare, Phil, and SPhil samples, we have fabricated and studied a textured sample which differs from SPhil by not applying the organosilane layer. That allows us to distinguish the roles of the oxide layer, organosilane, and the developed surface morphology. The analysis of the obtained data given in Table 2, allows us to conclude that the organosilane layer provides additional protection and reduces the corrosion current for samples contacted to corrosive solution for 2 h by approximately three times. At the same time, the developed surface area of the textured samples increases the corrosion current by order of magnitude. The latter also holds after 24 h of contact with NaCl. At the same time, prolonged exposure to corrosive media leads to pitting corrosion, compromising the organosilane layer and resulting in similar corrosion currents for both coated and uncoated samples. Through this, the highest corrosion resistance was demonstrated by the Phil coating, which enjoyed the benefits of low apparent/real surface area ratio and an additional barrier layer.

## 4. Summary and Conclusions

In this study, our primary objective was to evaluate how hydrophilic and superhydrophilic organosilane coatings affect corona discharge performance in high-voltage wires. We also examined secondary properties—specifically ice adhesion and corrosion resistance—to ensure that any improvements in corona mitigation would not compromise broader operational requirements. Bare wires served as the baseline for comparison, allowing us to assess whether the application of the coatings is expedient for improving overhead power transmission lines performance.

1. Notably, the corona protection ability of the hydrophilic organosilane coating proved comparable to that of the superhydrophilic coating. In particular, the hydrophilic variant reduced corona discharge power losses by up to 25% under initial exposure, achieving even greater reductions, of around 60%, after prolonged exposure under rainy conditions.

2. Ice adhesion tests showed that the hydrophilic coating maintained values within 10% of those for the bare wire, demonstrating consistent shear ice adhesion strength under icing conditions. In contrast, the superhydrophilic coating displayed substantially higher ice adhesion, potentially limiting its suitability in cold climates.

3. Corrosion testing revealed that the hydrophilic coating achieved the lowest initial corrosion current among all tested samples, reducing corrosion rates by approximately threefold compared to the bare wire. Meanwhile, the superhydrophilic coating exhibited a slightly higher corrosion rate than the bare wire, as its increased surface area outweighed the barrier properties of the organosilane and thick textured alumina layer.

Overall, the hydrophilic organosilane coating emerges as the most balanced solution, offering significant corona discharge reduction while preserving acceptable levels of ice adhesion and corrosion resistance. These qualities make it a highly suitable candidate for improving the durability and efficiency of high-voltage transmission lines under diverse environmental conditions.

## Figures and Tables

**Table 1:** Shear ice adhesion strength of bare, hydrophilic, and superhydrophilic coatings depending on different de-icing conditions.

Sample	Shear Adhesion Strength, kPa		
	Sample’s Angular Acceleration 4.35 rad/s^2^	15 h of Relaxation + Sample’s Angular Acceleration 4.35 rad/s^2^	Sample’s Angular Acceleration 0.54 rad/s^2^
Bare	92 ± 18	81 ± 29	54 ± 7
Phil	84 ± 17	85 ± 33	58 ± 17
SPhil	265 ± 45 *	232 ± 36 *	206 ± 73 *

* for most of the samples, the contact was destroyed by a cohesive mechanism, and they were excluded from the calculation.

**Table 2:** Corrosion currents for bare, hydrophilic, superhydrophilic, and textured (without organosilane layer) coatings after exposure to corrosive media for different timespans.

Sample	Time of Contact with 0.5 M NaCl, h	Corrosion Current, A/cm^2^
Bare	2	(7.6 ± 0.8) × 10^−7^
Phil		(2.6 ± 0.5) × 10^−7^
SPhil		(3.9 ± 0.7) × 10^−6^
Textured		(9.1 ± 1.1) × 10^−6^
Bare	24	(9.4 ± 0.5) × 10^−7^
Phil		(7.4 ± 0.5) × 10^−7^
SPhil		(5.1 ± 0.8) × 10^−6^
Textured		(11.8 ± 1.2) × 10^−6^

## Data Availability

The original contributions presented in the study are included in the article/Supplementary Materials. Further inquiries can be directed to the corresponding author.

## Supplementary Materials

The following supporting information can be downloaded at: https://www.mdpi.com/article/10.3390/ma18020254/s1, Figure S1: Photography of the high-voltage corona discharge experimental setup under normal light; Figure S2: SEM image of laser textured alumina prior to application of organosilane coating; Figure S3: EDX element map of Phil coating prior to corona discharge exposure. Carbon and Silicon is distributed over whole surface with bright spots of higher concentration of these elements in the depressions of the relief; Figure S4: EDX element map of Phil coating after corona discharge exposure; Figure S5: EDX element map of SPhil coating prior corona discharge exposure; Figure S6: EDX element map of SPhil coating after corona discharge exposure; Table S1: Element composition of Phil and SPhil coatings prior and after corona discharge exposure, atomic %; Table S2: Contact angles for coatings studied. Note, that for Bare and Hydrophilic coatings, angle was determined by sessile drop method, while for superhydrophilic coating, a captive bubble approach was used.
