# Supplementary material for: Efficient Corona Suppression Coatings and Their Behavior in Corrosive and Icy Environments
Source: Materials (Basel). 2025 Jan 9;18(2):254. doi: 10.3390/ma18020254 (PMC11766518; doi:10.3390/ma18020254)
Supplement: Supplementary file 1 [file materials-18-00254-s001.zip › materials-3290954-supplementary.pdf]

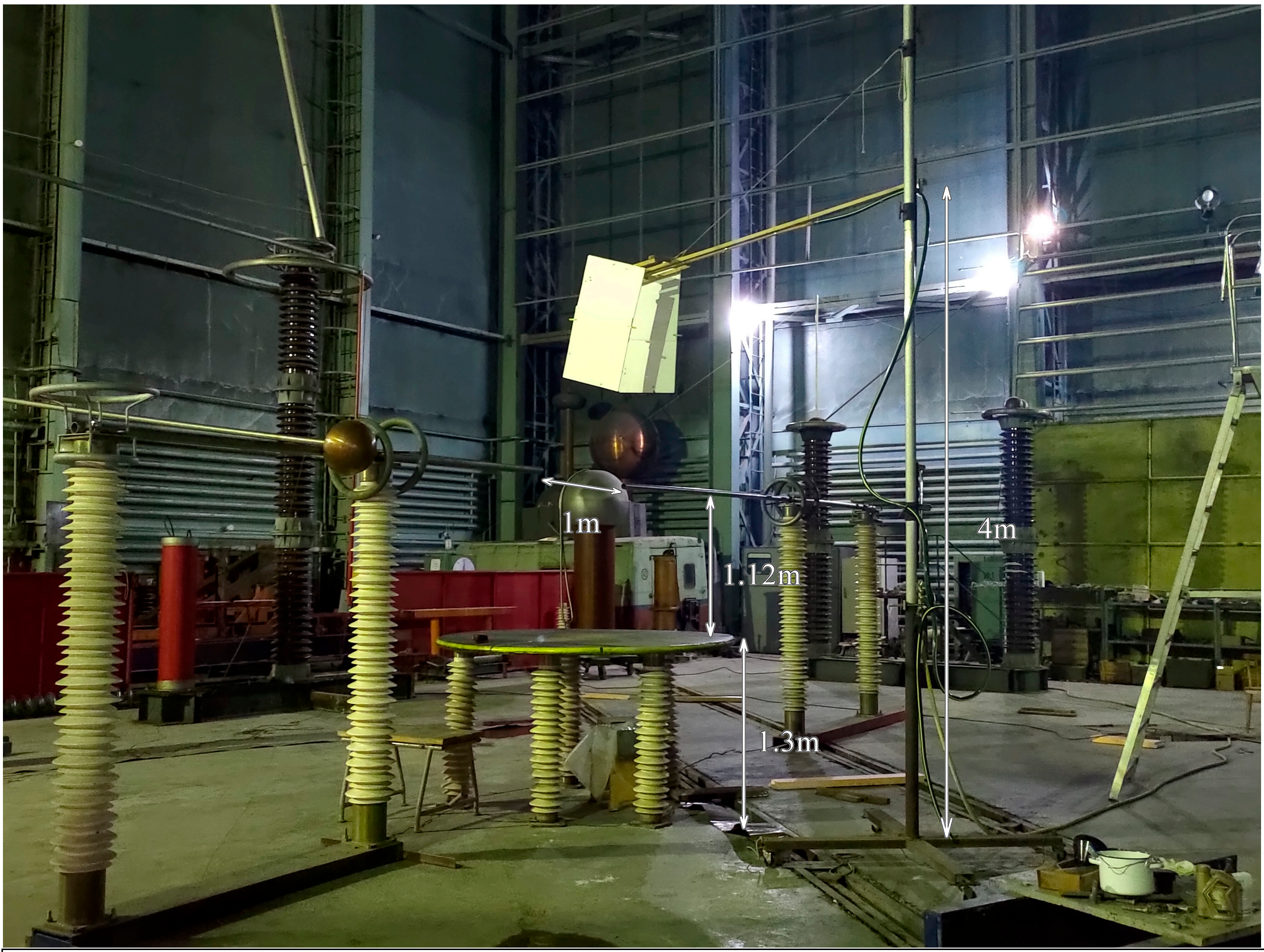

Figure S1. Photography of the high-voltage corona discharge experimental setup under normal light.

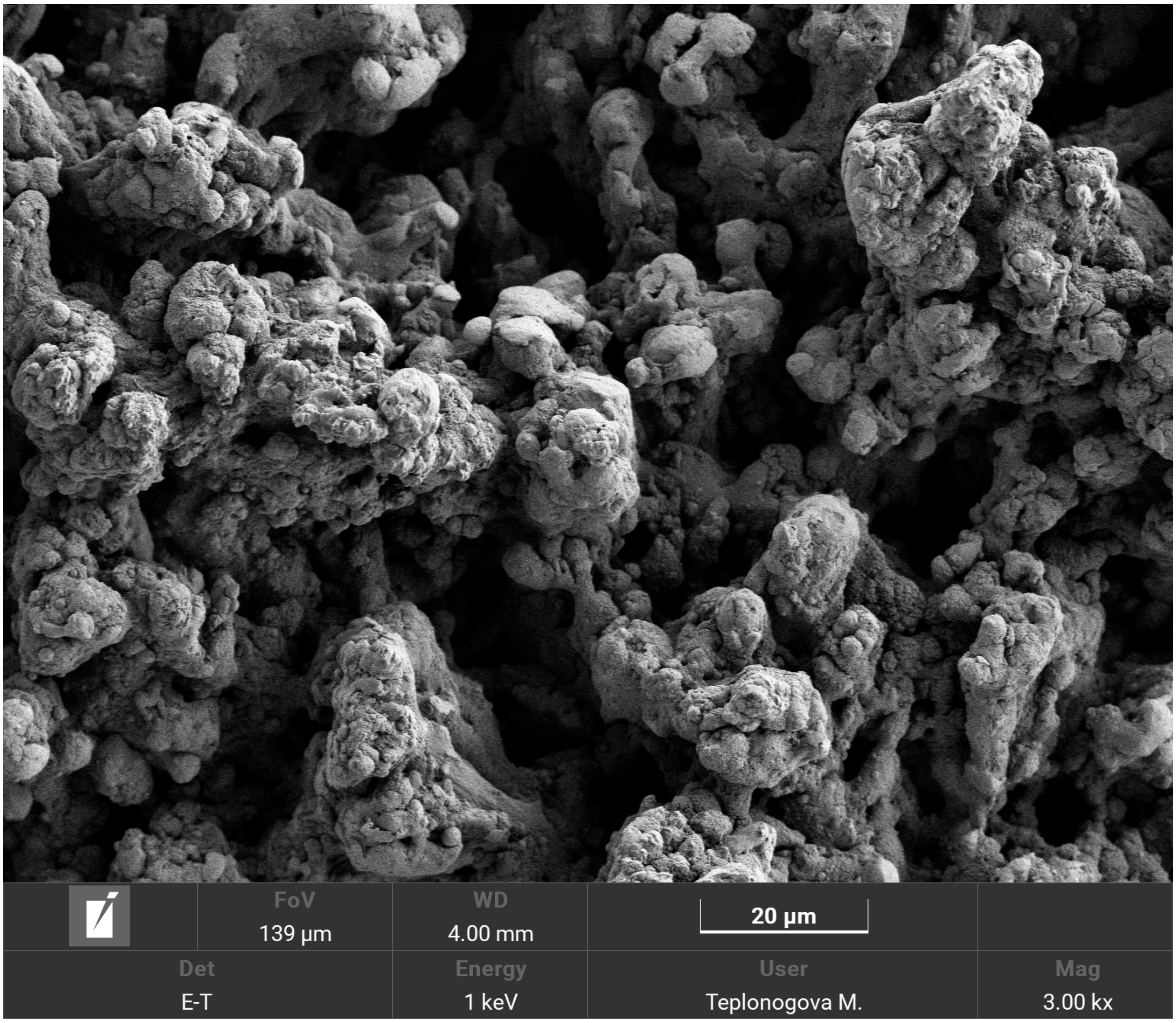

Figure S2. SEM image of laser textured alumina prior to application of organosilane coating.

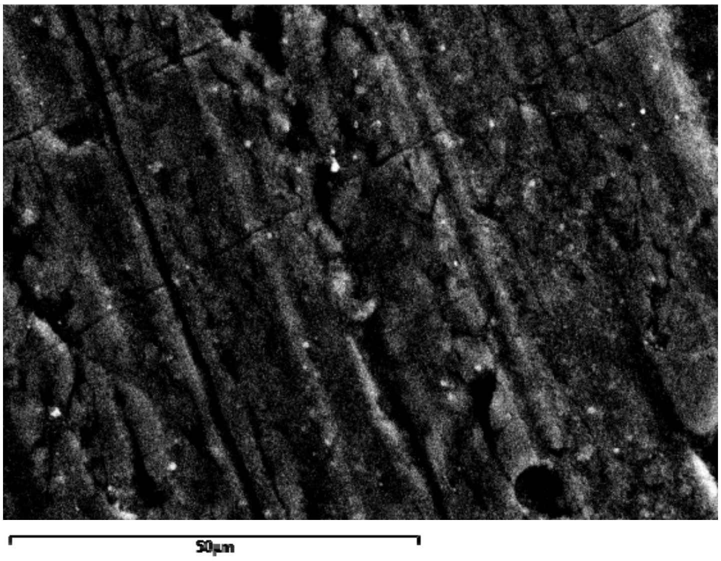

O

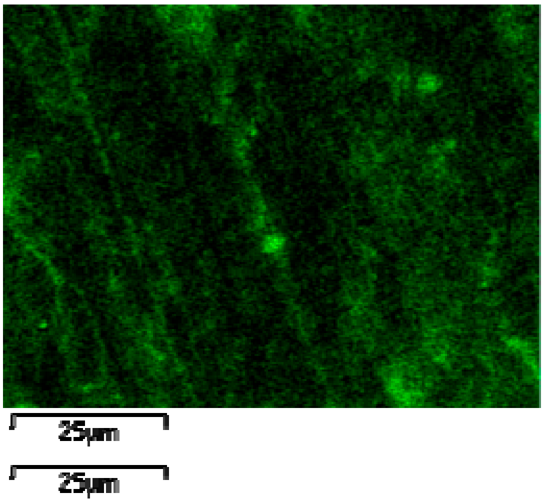

Figure S3. EDX element map of Phil coating prior to corona discharge exposure. Carbon and Silicon is distributed over whole surface with bright spots of higher concentration of these elements in the depressions of the relief.

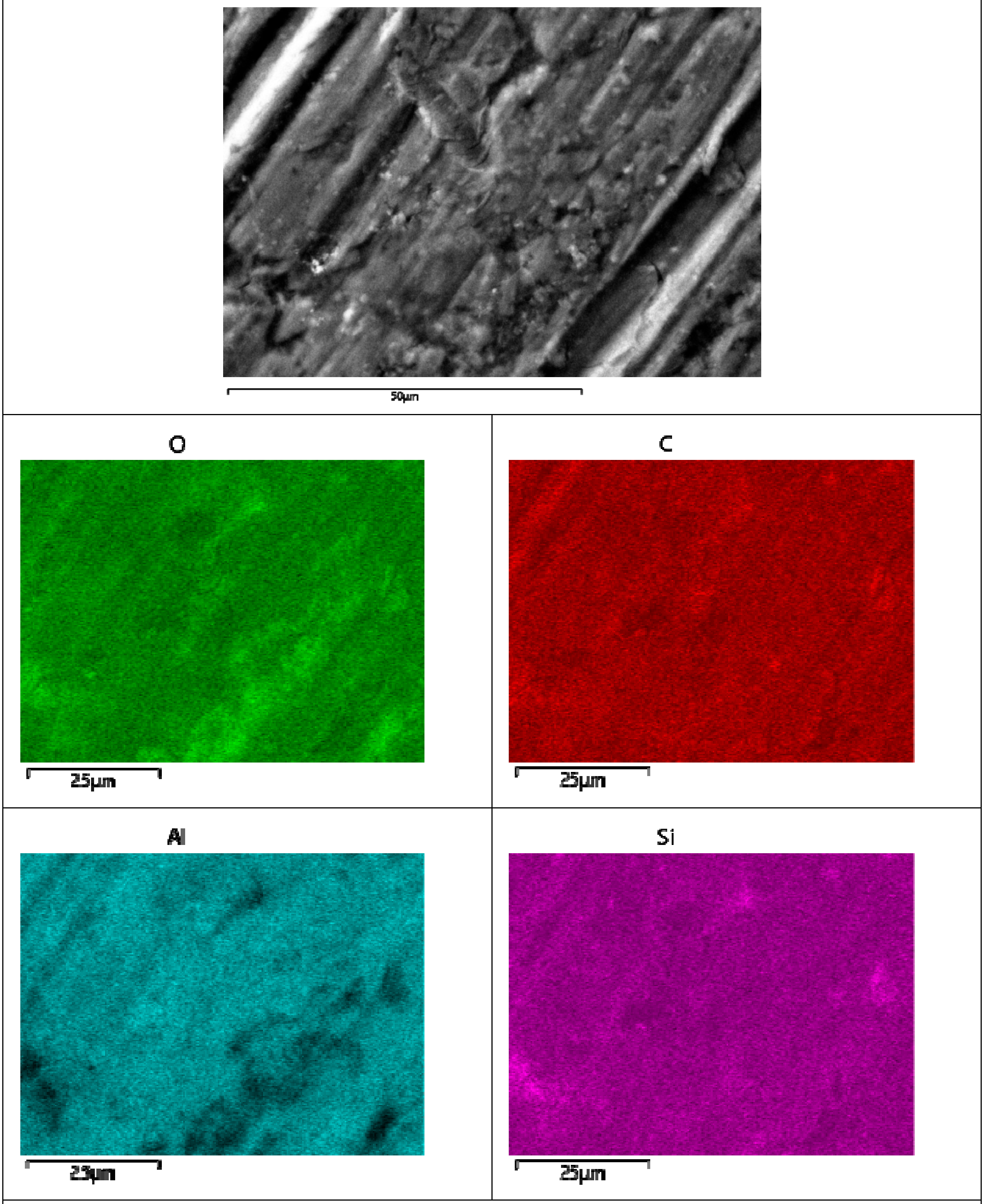

Figure S4. EDX element map of Phil coating after corona discharge exposure.

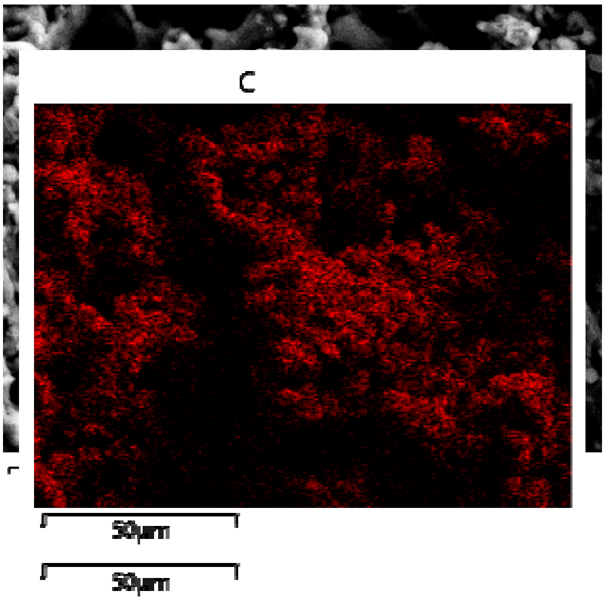
C
50µm
50µm

Figure S5. EDX element map of SPhil coating prior corona discharge exposure.

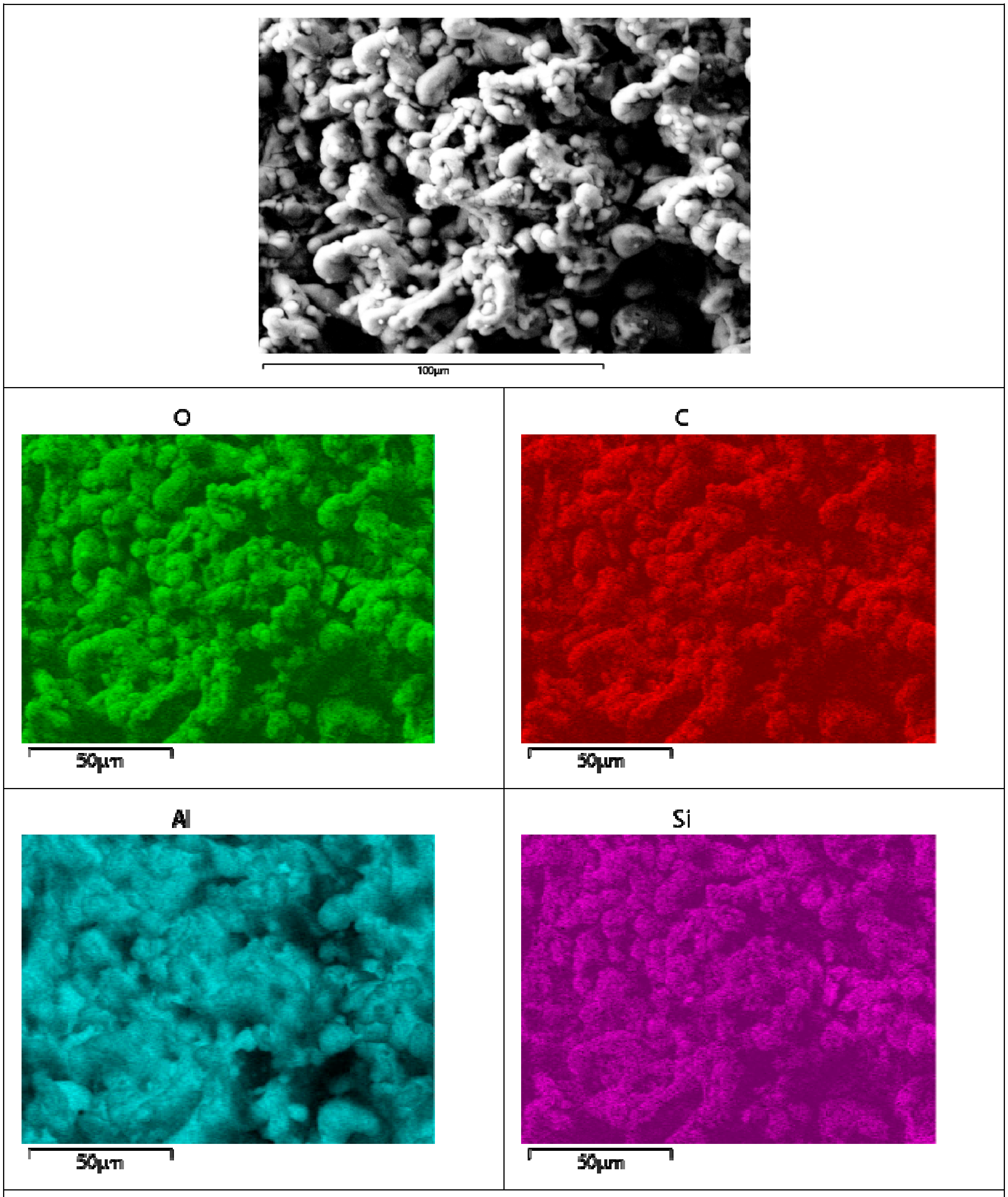

Figure S6. EDX element map of SPhil coating after corona discharge exposure.

Table S1. Element composition of Phil and SPhil coatings prior and after corona discharge exposure, atomic %

Sample	Al	Si	C	O	Mg	Mn	Fe
Phil_prior	67 ± 10	-	17 ± 5	8 ± 6	5 ± 5	0.4 ± 0.6	-
SPhil_prior	44 ± 9	0.7 ± 0.1	19 ± 4	32 ± 9	2.6 ± 0.4	0.1 ± 0.1	0.1 ± 0.1
Phil_after	47 ± 8	1.0 ± 0.3	42 ± 5	10 ± 3	-	-	-
SPhil_after	35 ± 8	2.1 ± 0.6	35 ± 4	29 ± 6	-	-	-

Table S2. Contact angles for coatings studied. Note, that for Bare and Hydrophilic coatings, angle was determined by sessile drop method, while for superhydrophilic coating, a captive bubble approach was used.

Sample	Bare	Hydrophilic organosilane coating	Superhydrophilic organosilane coating
Water Contact Angle,°	50 ± 3°	50.9 ± 0.8°	10.3 ± 0.5°
Surface layer composition	thin alumina oxide film	organosilane film	thick textured alumina oxide layer covered by organosilane film
